# Inhibition of PI3K-Akt Signaling Blocks Exercise-Mediated Enhancement of Adult Neurogenesis and Synaptic Plasticity in the Dentate Gyrus

**DOI:** 10.1371/journal.pone.0007901

**Published:** 2009-11-19

**Authors:** Elodie Bruel-Jungerman, Alexandra Veyrac, Franck Dufour, Jennifer Horwood, Serge Laroche, Sabrina Davis

**Affiliations:** 1 CNRS, UMR 8620, Orsay, France; 2 Université Paris-Sud, Laboratoire de Neurobiologie de l'Apprentissage, de la Mémoire et de la Communication, UMR 8620, Orsay, France; INSERM U862, France

## Abstract

**Background:**

Physical exercise has been shown to increase adult neurogenesis in the dentate gyrus and enhances synaptic plasticity. The antiapoptotic kinase, Akt has also been shown to be phosphorylated following voluntary exercise; however, it remains unknown whether the PI3K-Akt signaling pathway is involved in exercise-induced neurogenesis and the associated facilitation of synaptic plasticity in the dentate gyrus.

**Methodology/Principal Findings:**

To gain insight into the potential role of this signaling pathway in exercise-induced neurogenesis and LTP in the dentate gyrus rats were infused with the PI3K inhibitor, LY294002 or vehicle control solution (icv) via osmotic minipumps and exercised in a running wheel for 10 days. Newborn cells in the dentate gyrus were date-labelled with BrdU on the last 3 days of exercise. Then, they were either returned to the home cage for 2 weeks to assess exercise-induced LTP and neurogenesis in the dentate gyrus, or were killed on the last day of exercise to assess proliferation and activation of the PI3K-Akt cascade using western blotting.

**Conclusions/Significance:**

Exercise increases cell proliferation and promotes survival of adult-born neurons in the dentate gyrus. Immediately after exercise, we found that Akt and three downstream targets, BAD, GSK3β and FOXO1 were activated. LY294002 blocked exercise-induced phosphorylation of Akt and downstream target proteins. This had no effect on exercise-induced cell proliferation, but it abolished most of the beneficial effect of exercise on the survival of newly generated dentate gyrus neurons and prevented exercise-induced increase in dentate gyrus LTP. These results suggest that activation of the PI3 kinase-Akt signaling pathway plays a significant role via an antiapoptotic function in promoting survival of newly formed granule cells generated during exercise and the associated increase in synaptic plasticity in the dentate gyrus.

## Introduction

It is well accepted that cell proliferation and neurogenesis continue to occur in selected brain regions of the adult brain, notably the subgranular zone of the dentate gyrus (DG) and the subventricular zone of the lateral ventricles [1]. Different forms of physiological and pathological conditions can promote neurogenesis, such as exercise [2] and environmental enrichment [3]–[5] and injurious circumstances such as ischaemia or seizures [1].

Physical exercise, in addition to promoting hippocampal neurogenesis, is also known to improve cognitive functions in humans and rodents [6]–[7] and to contribute to the preservation of cognitive performance in ageing and neurodegenerative disorders such as Alzheimer's disease [8]–[9]; both of which are associated with deficient hippocampal neurogenesis [10]. In rodents, exercise exerts a beneficial effect on spatial learning [2], [11]–[12] and some studies have associated the exercised-induced improvement in learning with its ability to promote neurogenesis and to facilitate long-term potentiation (LTP) in the dentate gyrus [13]–[14]. These findings, together with the demonstration that immature dentate granule cells (DGCs) are more responsive to LTP than mature cells [15]–[16], suggests that the facilitation of LTP following exercise may be a direct outcome of the increased production of young dentate gyrus cells induced by exercise.

To date, however, the potential mechanisms that subserve the beneficial effects of exercise-induced neurogenesis remain largely unknown. A number of genes and proteins have been shown to be regulated by exercise [17]–[18]; some of which are also associated with neurogenesis. Most notably, are the growth factors BDNF, IGF, FGF-2 and VEGF that activate signalling pathways such as MAPK/ERK and PI3K-Akt [19]. Recently, Chen and Russo-Neustadt [20] reported activation of the serine/threonine kinase Akt in the whole hippocampus after exercise. The PI3K-Akt signalling pathway is potentially implicated in a number of different functions, such as glucose metabolism, protein synthesis, receptor insertion, cytoskeletal reorganisation and cell proliferation, however, it is most commonly associated with cell survival by inhibiting the activation of proapoptotic proteins and transcription factors [21]–[22]. Given the role of the PI3K-Akt signalling pathway in cell survival, our aim in these experiments was to investigate the potential role of this signalling pathway in exercise-induced neurogenesis in the adult dentate gyrus and to measure the extent to which the modulation of adult neurogenesis affects LTP in this structure. We first showed that following exercise in a running wheel, Akt is hyperphosphorylated and in turn several of its downstream targets, FOXO, BAD and GSK3β known for their antiapoptotic functions, are phosphorylated by Akt in the dentate gyrus. We also showed that inhibiting PI3K by infusion of LY294002 prevents exercise-induced phosphorylation of these proteins and stems exercise-induced neurogenesis in the dentate gyrus without affecting cell proliferation. Finally, as a functional assay, we induced LTP in the dentate gyrus two weeks following the exercise period. LTP was facilitated in parallel with the increase in neurogenesis in control runners and inhibition of the PI3K completely blocked facilitation of LTP in association with the reduction in the number of surviving cells. Taken together, these data represent a step forward in the identification of the pro-survival role of the PI3K-Akt pathway in regulating neurogenesis in the adult dentate gyrus.

## Materials and Methods

### Animals

Young male adult Sprague-Dawley rats (10–12 weeks; Charles River, France) were housed singly in temperature-controlled conditions with a 12 hr light/dark cycle (lights on: 8:00 AM) following surgery. They had access to food and water *ad libitum*. Experimental procedures were conducted in accordance with recommendations of the European Union (86/609/EEC) and the French National Committee (87/848).

### Surgical Procedure

Standard surgical procedures were conducted to implant cannulae attached to osmotic mini-pumps. Rats were anaesthetized with sodium pentobarbital (60 mg/kg), supplemented throughout surgery as required. Cannulae, attached to the minipump via a short length of tubing, were slowly lowered into the left ventricle (Bregma −0.9 mm; ML 1.3 mm; DV from brain surface 3.4 mm) and fixed in place with dental acrylic. A small subcutaneous pocket was then opened up between the shoulder blades of the rat to fit the mini-pump in place. The skin overlaying the skull was sutured and topical antiseptic (exocptoplix) was applied to the wound. Rats were then returned to home cages to recover for two days during which time they were handled before starting habituation to the running wheel.

### Drugs and Drug Delivery

Twenty-eight-day osmotic mini-pumps (Model 2004; Alzet) with a pump rate of 0.25 µl/hour were used. Pumps were loaded either with 30% DMSO and aCSF for control rats or the PI3K inhibitor, LY294002 (5 mM dissolved in 30% DMSO and aCSF (Na, 150 mM, K, 3 mM; Ca, 1.4 mM; Mg, 0.9 mM; Cl, 99 mM, Ozyme, France); made up in pyrogen-free, sterile water according to the instructions from Alzet). BrdU (Sigma) was injected intraperitoneally (i.p. 100 mg/kg; dissolved in 0.9% NaCl and 0.007% NaOH (1N)) on the last three days of exercise.

### Electrophysiology

Twelve to fourteen days following the end of the exercise period, rats (Naïve, n = 8; DMSO, n = 7; LY294002, n = 8) were anesthetized with urethane carbamate (1.5 mg/kg), placed in a stereotaxic frame, and maintained at a constant body temperature of 37°C. Pumps and cannula were left in place and electrodes were implanted in order to induce LTP at perforant path-dentate gyrus synapses. The stimulating electrode was placed in the angular bundle of the perforant path (bregma −8.0 mm, 4.2 mm from midline, 2.6 mm depth) and the recording electrode in the DG (bregma −4.2 mm, 2.5 mm from midline). Implantation of electrodes was made under electrophysiological guidance as describe previously [23]. Low-frequency test pulses (100 µsec, 0.033 Hz) were delivered by a photically isolated constant current unit. After responses had stabilized, a 20-min baseline was recorded, followed by a tetanus to induce LTP, consisting of 6 series, 2 min apart, of 6 bursts of high-frequency stimulation (400 Hz, 20 ms) at 10 sec intervals. This protocol was chosen as it reliably induces saturated LTP in the dentate gyrus *in vivo* [24]. Evoked responses to test pulses were recorded for a minimum of 2 h following the tetanus to ensure that L-LTP was induced. Evoked responses were stored for off-line analysis of the EPSP slope and the population spike. Analysis of variance and Fisher PLSD post hoc analysis were conducted on the mean of the last 15 minutes of recording of the slope of the EPSP and the population spike, the mean basal EPSP values and the intensity required to evoke the response.

### Immunohistochemistry for BrdU Labeling and Cell Counting

Rats were anaesthetized with an overdose of sodium pentobarbital and transcardially perfused with 0.1 M phosphate buffer (PB) followed by 4% paraformaldehyde in 0.05 M PB, at 4°C. The brains were left in the fixative overnight, and then transferred to 30% sucrose. Coronal sections (30 µm) were serially cut using a cryostat and stored in cryoprotectant at −20°C until being processed for BrdU labeling. Peroxide immunolabeling was performed as previously described [25]. Sections throughout the DG were hydrolyzed with 2N HCl at 37°C for 40 min and incubated overnight in primary mouse monoclonal anti-BrdU (1∶1000, MAB 3424; AbCys, France) in PB containing 5% BSA, 0.5% Triton X-100 and 5% normal goat serum. Sections were incubated in biotinylated goat anti-mouse antiserum (1∶200), then in avidin-biotin-peroxidase complex (1∶100; Vectastain Elite Kit, Vector Laboratories) and were reacted for peroxidase detection (DAB kit, Vector Laboratories). In order to measure the area of the DG, sections were counterstained with Nuclear fast red (Vector Laboratories). For immunohistofluorescence, sections were hydrolyzed as described above, followed by overnight incubation in monoclonal rat anti-BrdU (1∶400, OBT0030; AbCys). Sections were incubated in biotinylated goat anti-rat antiserum (1∶400; Vector Laboratories), then in streptavidin Alexa-568 (1∶800; Invitrogen, Eugene, OR). Sections were then incubated overnight in mouse monoclonal anti-NeuN (1∶500; Chemicon), in Alexa-488 goat anti-mouse highly cross-adsorbed antiserum (1∶200; Molecular Probes) and counterstained with DNA dye bisbenzimid (Hoechst 33342, Sigma; 1 ug/mL). Antibodies were tested with the appropriate negative controls (reciprocal omission of primary and secondary antibodies).

Stereological quantification of BrdU-labeled nuclei in the DG was conducted bilaterally in every 6^th^ section to assess cell survival and every 4^th^ section to assess cell proliferation as described previously [5], [25]. To avoid oversampling errors, nuclei intersecting the uppermost focal plane were excluded. Absolute numbers of BrdU-labeled cells were obtained by multiplying BrdU-positive cell density by the reference volume. For double-labeling, percentages of BrdU-labeled nuclei co-expressing NeuN were determined by analyzing 100 randomly selected BrdU-labeled nuclei throughout the DGC layer and subgranular zone (SGZ) of dorsal DG using a Zeiss confocal microscope (Oberkochen, Germany). Absolute numbers of new neurons (BrdU-NeuN) were estimated by multiplying the absolute numbers of BrdU cells by the percent of co-localisation for those two markers. BrdU-positive nuclei were analyzed (63x oil objective) in their entire *z*-axis (0.5 µm steps) and were rotated in orthogonal planes (*x–y*) to verify double-labeling and exclude false double-labeling caused by overlay of signals from different cells. Analyses were performed in sequential scanning mode to rule out cross-bleeding between detection channels.

### ImmunoWestern Blotting

Rats were killed by decapitation and their brains removed rapidly on ice. The DG was dissected out of the hippocampus and frozen in liquid nitrogen. Tissue was homogenized in a lysis buffer and centrifuged at 15493 *g* for 20 minutes. A Bradford protein assay was used to assess total protein levels and all samples were equalized to the same protein content. Immunoblots were prepared following previously described protocols [23], [26]. Following electrophoresis, proteins were transferred to nitrocellulose membranes, blocked for 1 h at room temperature in 5% BSA and incubated overnight at 4°C in primary antibodies. Membranes were then rinsed and incubated with secondary antibody (horseradish peroxidase-conjugated anti-rabbit IgG, Amersham) for 1 h and then reacted with electrogenerated chemiluminescence, apposed to film and developed by hand. Membranes were then stripped of antibodies and probed with non-phospho antibodies. Optical density of protein bands on film was analyzed with GENETOOLS analysis software (GeneGenius Gel Documentation System, UK). Phospho antibodies were pAkt-Ser473 (1∶2000) and Thr308 (1∶2000), pBAD (1∶1000), pGSK3β-Ser9 (1∶3000), FOXO1-Ser256 (1∶2000) and pERK (Thr183/Tyr185) (1∶2000). Concentrations for the corresponding non-active antibodies were: AKT (1∶1000), BAD (1∶1000), GSK3β (1∶3000), FOXO1 (1∶1500) and ERK (1∶3000). All primary antibodies to Akt and downstream target proteins were purchased from Cell Signalling (Ozyme, France). Western blotting analysis of KI-67 (Abcam, France), a reliable endogenous marker of cell proliferation present throughout the entire cell cycle phase except G0 [27], was carried out using discontinuous (3–8%) SDS-PAGE precast gels (Invitrogen). Proteins were transferred to nitrocellulose and treated as above with the exception that membranes were blocked and anti-KI-67 (1∶1000, from Abcam, France) was diluted to concentration in milk (Biorad, France). At least 3 replicates were processed for each protein assay. Optical density values from total proteins were analyzed to determine whether there was any change in density and if not, phospho-proteins were normalized to these values and results were averaged per rat. These were then normalized to the mean of the naïve group for analyses.

### Experimental Protocol

Three groups of rats were used throughout the experimental procedure: naïve rats that received no treatment or exercise; rats implanted with minipumps containing vehicle solution, DMSO that underwent the exercise regime, and rats implanted with minipumps containing the PI3K inhibitor, LY294002 that underwent the exercise regime. Rats were handled for 5 days before and 2 days after surgery. On the third day after surgery they were habituated to a running wheel (28 cm diameter, Campden Instruments). This started by placing the rat in the wheel in a fixed position and then slowly over the next 4 days they were habituated to turning the wheel at will and then to forced running. We chose a force run protocol rather than free running as we wished to clamp the distance run per day across animals. The running protocol consisted of 2, one-hour sessions (AM and PM), attempting to maintain the same speed across all animals. The number of revolutions run by each rat was calculated as km/day. All rats were injected with the birthdating marker BrdU on the three last days of the running session.

**Figure 1:**
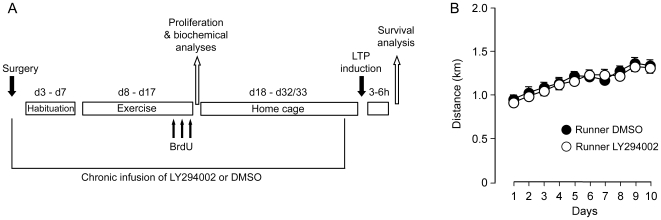
Experimental design and exercise performance. A, Schematic representation of the experimental protocol. B, Distance run in kilometres over the 10-day exercise period. Each point represents the combined distance covered each day for the two-hour session. Both groups started with the same number of kilometers (DMSO, black circles: DMSO: 0.94±0.05 km; LY294002, open circles: 0.89±0.05 km) and increased their distance run over the 10 days (DMSO: 1.34±0.07 km; LY294002: 1.31±0.06 km).

To assess whether exercise regulated Akt and downstream antiapoptotic targets, increased cell proliferation and whether this was affected by inhibiting PI3K, rats (n = 8 per group) were killed 10 minutes following the last exercise session. In half of the rats (n = 4 per group) DG tissue was removed for immunowestern blotting of Akt and its downstream target proteins, FOXO1, GSK3β and BAD and the endogenous marker of proliferation, KI-67. As PI3K under certain conditions can interact with the MAPK/ERK pathway, we also assessed activation of phospho-ERK. In the other half of the rats (n = 4 per group) we conducted stereological counting of BrdU-labeled cells. On the last three days of exercise a single injection of BrdU was given i.p. in between the two exercise sessions and rats were perfused 2 hours after the last BrdU injection. Finally, to examine the effect of inhibiting PI3K in the survival of newborn cells and on synaptic plasticity, following the 10 days of exercise, rats (DMSO runners  = 7; LY294002-treated runners  = 8; naïve  = 8) were returned to their home cages for between 14 to 16 days; by which time the pumps' content would be spent. At this point we induced LTP as a functional measure of the effect of running synaptic plasticity. At the end of the recording session, rats were perfused and brains prepared for BrdU and NeuN immunohistochemistry (see Fig. 1A). All analyses were conducted using Analysis of Variance (ANOVA) and Fisher Post Hoc Analyses with the probability set to 0.05.

## Results

During the 10 days of exercise, as we controlled the amount of running, DMSO and LY294002 treated rats from the three different experiments were pooled for analyses. All animals ran an almost identical number of kilometers per day with no difference between rats treated with DMSO (n = 18) and those treated with LY294002 (n = 19) across days (F(1,35) = 0.175; p = 0.678; Fig. 1B) or in terms of total km run (F(1,35) = 0.277; p = 0.603); both groups, however showing a comparable and significant increase in running over the 10 days (F(1,9) = 28.8; p = 0.0001).

### Exercise Activates the PI3K-Akt Signaling Pathway

**Figure 2:**
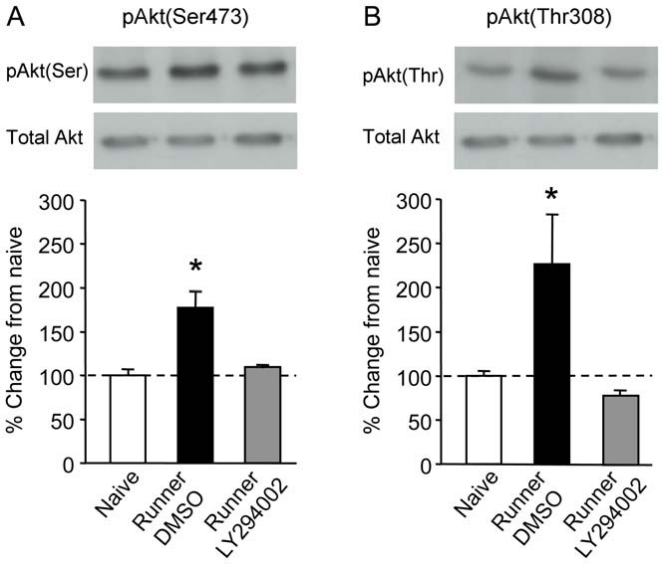
Regulation of pAkt in the dentate gyrus in exercising rats. A, Phosphorylation of Akt at serine site 473 and B, Akt phosphorylation at threonine site 308 in DMSO-treated runners (Black bars, n = 4) is significantly greater than both LY294002-treated runners (Grey bars, n = 4) and naïve rats (White bars, n = 4). Histograms represent the percent change in pAkt normalized to naïve controls. PhosphoAkt levels at both sites in LY294002-treated runners are not significantly different from naïve rats. Sample blots for each group are presented in upper panels. No change in total Akt was observed.

**Figure 3:**
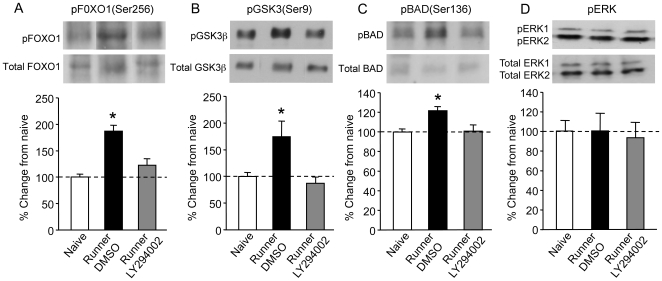
Regulation of downstream target proteins of Akt in the dentate gyrus following exercise. A, Phosphorylation of FOXO1(Ser256), B, GSK3β(Ser9), and C, BAD(Ser136) in the three groups (n = 4 per group). For all three proteins, DMSO-treated runners (Black bars) showed a significantly greater level of phosphorylation of the three Akt target proteins than either the naïve rats (White bars) or LY294002-treated runners (Grey bars). LY294002-treated runners show no significant increase in phosphoprotein levels compared with naïve rats, although there was a slight increase in pFOXO1. 3D. Exercise does not lead to phosphorylation of ERK. DMSO-treated runners (Black bars, n = 4) show no difference in phosphoERK levels compared with naïve rats (White bars, n = 4). Neither does inhibition of PI3 kinase effect levels of ERK, as there is no difference between LY294002-treated runners (Grey bars, n = 4) and DMSO-treated runners or naïve rats. Sample blots for each group are represented in the upper panel.

Regulation of Akt and several of its downstream targets was examined in the subgroup of rats killed 10 min following the last session of running. Firstly, there was no significant difference between groups in total levels for any of the proteins analysed (Akt (F(2,9) = 3.0; p = 0.105); ERK (F(2,9) = 3.61; p = 0.0706); FOXO1 (F(2,9) = 2.27; p = 0.16); GSK3β (F(2,9) = 1.54; p = 0.26); BAD (F(9,2) = 1.04; p = 0.393). Phosphoproteins were normalised to the total content of their respective proteins. Secondly, in exercised rats infused with DMSO (n = 4), Akt was hyperphosphorylated at both serine and threonine sites compared with naïve rats (n = 4) and exercised rats infused with LY294002 (n = 4) (Ser-473: (F(2,9) = 13.32; p = 0.002); Thr-308: (F(2, 9) = 4.9; p = 0.036); Fig. 2). Post hoc analyses showed that inhibiting PI3K activity abolished exercised-induced hyperphosphorylation of Akt, as LY294002-treated rats showed no difference in levels of pAkt at either site compared to naïve rats (p>0.05 in each case). Similar increases in phosphorylation of the downstream targets of Akt were observed with FOXO1-Ser256 (F(2,9) = 18.9, p = 0.00006), GSK3β-Ser9 (F(2,9) = 6.21; p = 0.02) and BAD-Ser136 (F(2,9) = 6.33; p = 0.0192) in DMSO runners, and the increased phosphorylation of these proteins was blocked in LY294002-treated runners (Post hoc comparisons with naïves: p>0.05 in each case; Fig. 3A–C). These data suggest that exercise induces full activation of Akt at both phosphorylation sites and a number of its downstream targets known to have a function in cell survival. We also analysed phosphorylation of ERK as PI3K can interact with proteins of the MAPK/ERK pathway; however we found that pERK was neither regulated by exercise nor attenuated by inhibition of PI3K (between-group difference: (F(2,9) = 0.009; p = 0.389); Fig. 3D).

### The Effect of PI3K-Akt Signaling on Exercise-Induced Cell Proliferation

**Figure 4:**
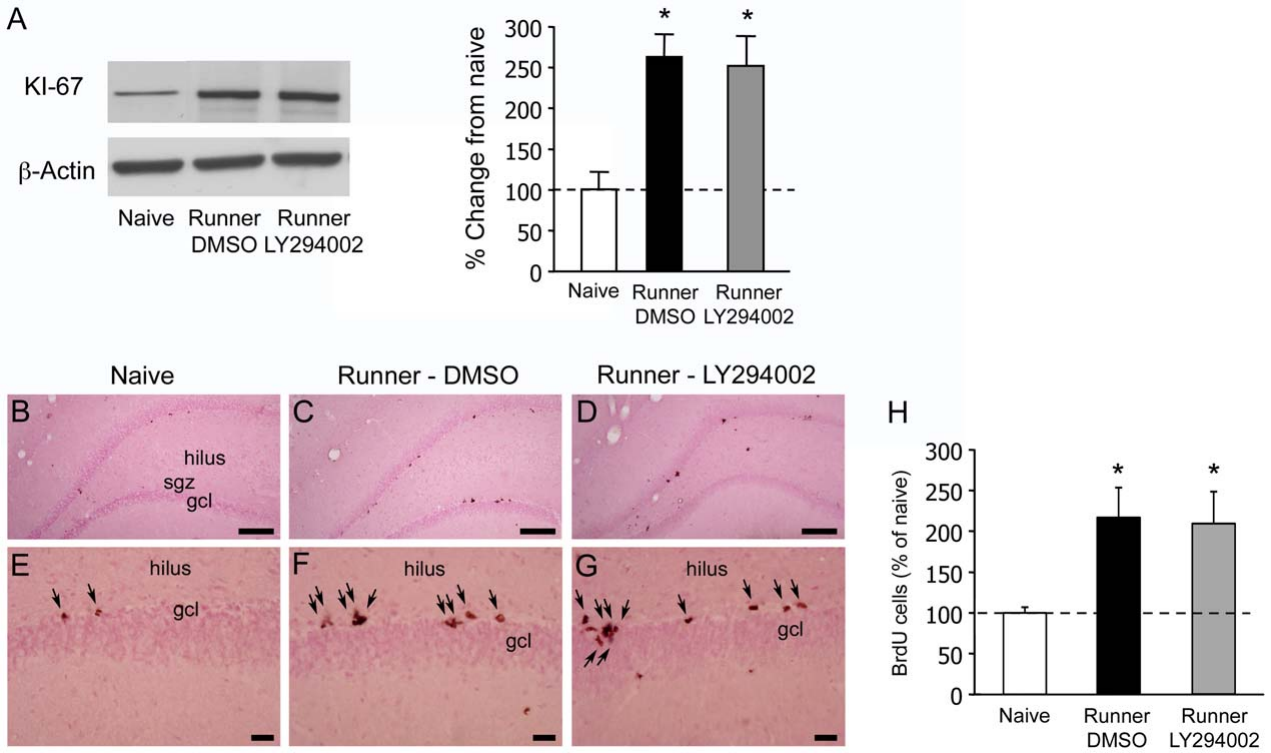
Effect of running and inhibition of the PI3K-Akt signaling pathway on dentate gryus cell proliferation. A. Expression of KI-67 in the dentate gyrus following exercise. KI-67 protein levels are increased in both DMSO-treated runner (Black bars, n = 4) and LY294002-treated runners (Grey bars, n = 4) compared with naïve rats (White bars, n = 4). Sample blots for each group are represented on the left panel. 4B–D, Representative light photomicrographs of Nuclear fast Red-stained sections shows distribution of BrdU immunoreactive nuclei in the dentate gyrus of (B) naïve, (C) DMSO-treated and (D) LY294002-treated runners; sgz, subgranular zone. E–G, Higher magnification illustrates increased numbers of proliferating cells in the sgz in (E) naïve rats, (F) DMSO-treated runners and (G) LY294002-treated runners. H, Quantitative data 2 hours after the last of three BrdU injections are expressed as the number of BrdU-cells (% of naives) to show comparable results with western blotting analyses of KI-67. Scale bars 200 µm (*B–D*), 100 µm (*E–G*).

First, we measured levels of KI-67, a protein that is exclusively expressed in proliferating cells and is associated with the regulatory mechanisms that drives the cell division cycle [28] using western blotting and found a comparable increase in the levels of KI-67 in exercising rats infused with DMSO or LY294002 compared with naïve rats ((F(2,9) = 9.49; p = 0.0061); Fisher PLSD post hoc analyses showed that both running groups were significantly increased compared with naïve rats, p<0.05; Fig. 4A). Secondly, we quantified the number of BrdU-labeled cells 2 hours following the last injection of BrdU and we found that running induced a large increase in clustered BrdU-labeled nuclei in the subgranular zone (Fig. 4B–G). We found no significant difference between the groups in the reference volume of the DG (F(2,9) = 2.162; p = 0.171, data not shown). Quantitative stereological analysis of BrdU-labeled cells revealed that the number of proliferating cells in the DG was increased by 115% in running–DMSO group and by 109% in running-LY294002 animals compared with naïve controls (Fig. 4H, naïve: 1357±94, n = 4; running-DMSO: 2929±497, n = 4; running-LY294002: 2835±531, n = 4; (F(2,9) = 4.336; p = 0.048); Fisher PLSD post-hoc comparison with naïves: both p values <0.05). Both results suggest that inhibition of the PI3K-Akt pathway during exercise does not affect exercise-induced proliferation of DG progenitor cells.

### Influence of PI3K-Akt Signaling on Survival of Newborn Neurons Generated during Exercise

**Figure 5:**
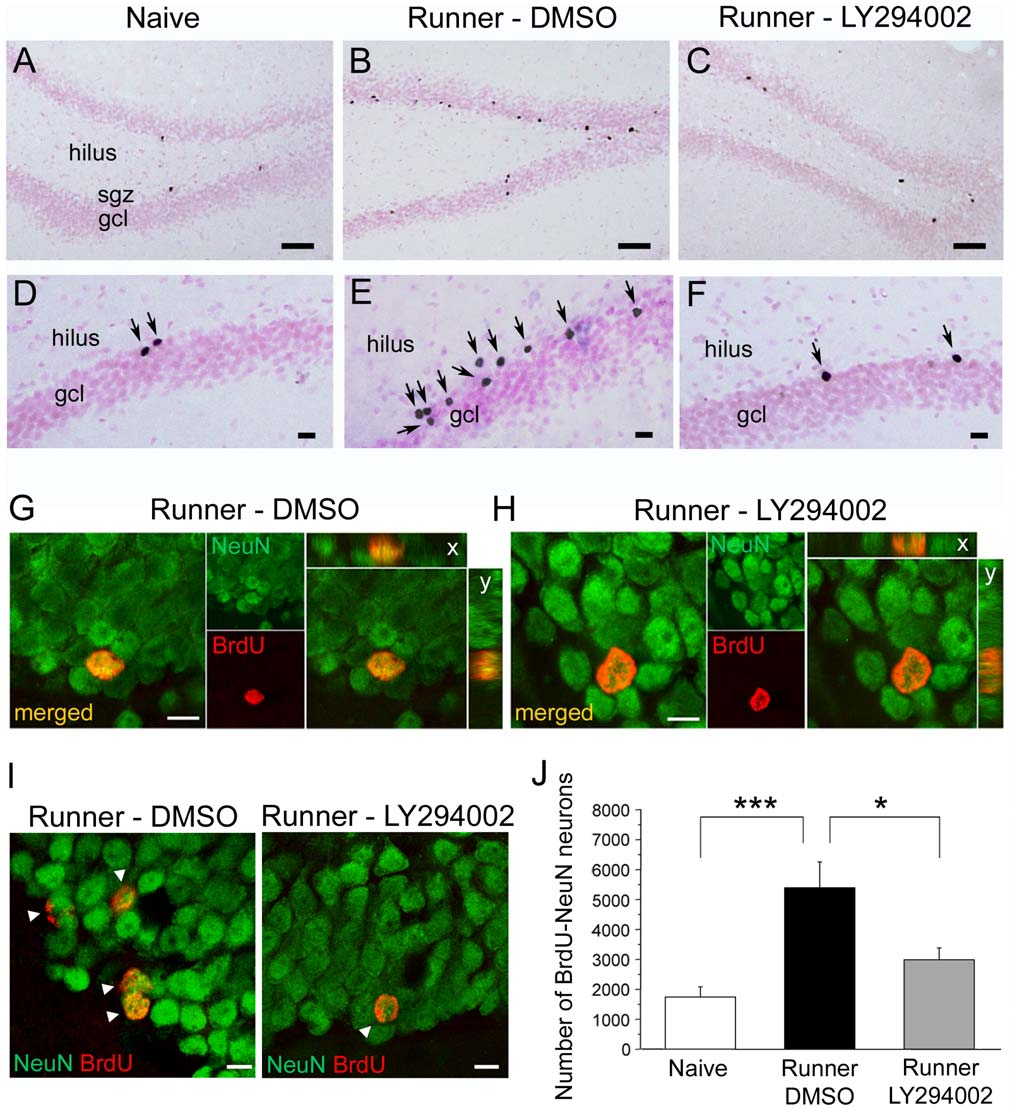
Effect of running and inhibition of the PI3K-Akt signalling pathway on dentate gryus neurogenesis. A–C, Representative light photomicrographs of Nuclear fast Red-stained sections shows distribution of BrdU immunoreactive nuclei in the dentate gyrus of (A) naïve, (B) DMSO-treated and (C) LY294002-treated runners (sgz, subgranular zone; gcl, granule cell layer). D–F, Higher magnification illustrates increased numbers of newborn cells in the gcl (arrows) in (E) DMSO-treated runners compared to (D) naïve rats and the reduction in the number of BrdU-labeled cells in (F) LY294002-treated runners. G,H, Confocal image of double-stained cells for BrdU (red) and NeuN (green) illustrates co-localisation in dentate granule cells (arrowheads) in representative sections from animals in the DMSO-treated (G) and LY294002-treated (H) runners. I, Representative confocal laser scanning microscope stack images depict cells double-labeled (merged) for BrdU (red) and NeuN (green) in the dentate gyrus in animals from both groups. BrdU-NeuN double-labeled cells are shown in x–y orthogonal planes and z-sectioning at 0.5 µm intervals (right) to confirm overlap of the two immunoreactions. J, Quantitative data 14–16 days after BrdU injections are expressed as the total number of BrdU-labeled cells (all groups, n = 5). Scale bars 100 µm (A–C), 25 µm (D–F) and 10 µm (G–I).

Groups of rats were returned to the home cage for 14–16 days following the last day of exercise; by which time the minipumps were spent. Newborn cells in the DG were then quantified by BrdU incorporation into nuclei of dividing cells (14–16 days after BrdU injections). BrdU-labeled nuclei were dark and round-shaped, frequently with the typical morphology of DGC nuclei (Fig. 5A–F). The comparison of the reference volume revealed that neither running nor LY294002 treatment had any significant effect (F(2,12) = 0.128; p = 0.881; Fisher's PLSD p>0.05 in each case, data not shown). There was a significant difference in the total number of BrdU-positive cells in the three groups (F(2,12) = 8.82; p = 0.004). As expected, running led to a large (∼3 fold) and significant increase in the number of BrdU-labeled cells in the dentate gyrus, compared with naïve controls (naïve: 2210±481, n = 5, Fig. 5A,D; running-DMSO: 6209±885, n = 5, Fig. 5B,E; Fisher PLSD post-hoc comparison p<0.001). This indicates that forced running, as voluntary running (2, 14), significantly increases neurogenesis in the dentate gyrus. In exercising rats that were infused with the PI3K inhibitor LY294002 the number of surviving BrdU-positive cells (3874±598, n = 5) was substantially reduced (Fig. 5C,F) compared with DMSO-treated animals (p<0.05; Fig. 5B,E) reaching a level close to, and not significantly different from naïve rats (p = 0.108; Fig. 5A,D).

The phenotype of BrdU-positive cells was examined by immunofluorescent double-labeling for BrdU and the neuron-specific marker NeuN. Confocal microscopy was used to count the number of double- and single-labeled BrdU-positive cells in the dentate gyrus (Fig. 5G,H). In all three experimental groups, co-localisation of BrdU with NeuN showed that the large majority of BrdU-positive cells expressed a neuronal phenotype (Fig. 5I). Despite the large increase in total number of BrdU-labeled cells in the runners, no change was found in the percentage of newborn cells expressing a neuronal phenotype compared with naïve rats, and similarly we found no evidence that LY294002 treatment affected the proportion of BrdU-NeuN co-expressing cells (naïve: 79.9±2.3%; running-DMSO: 86.1±2.1%; running-LY294002: 78.9±3.1%; (F(2,12) = 2.336; p = 0.139). Calculation of the absolute number of cells co-expressing BrdU and NeuN in the three groups confirmed the increase in the number of newborn neurons after running and the significant reduction in LY294002-treated rats (naïve: 1742.9±340.6; running-DMSO: 5405.2±864.0; running-LY294002: 2992±398.4; F(2,12) = 10.193; p = 0.003; Fig. 5J). Fisher PLSD post-hoc comparison showed the differences to be between naïve and DMSO runners (p<0.05) and DMSO runners and LY294002-treated runners (p<0.05). Thus, neither running nor inhibition of PI3K-Akt activity affected neuronal commitment of newly generated DGC's. In all, these results confirm that running promotes neurogenesis in the dentate gyrus and show that most of the beneficial effect of running on neurogenesis is abolished by inhibition of the PI3K-Akt signaling pathway.

### Exercise-Induced Neurogenesis and LTP in the Dentate Gyrus

**Figure 6:**
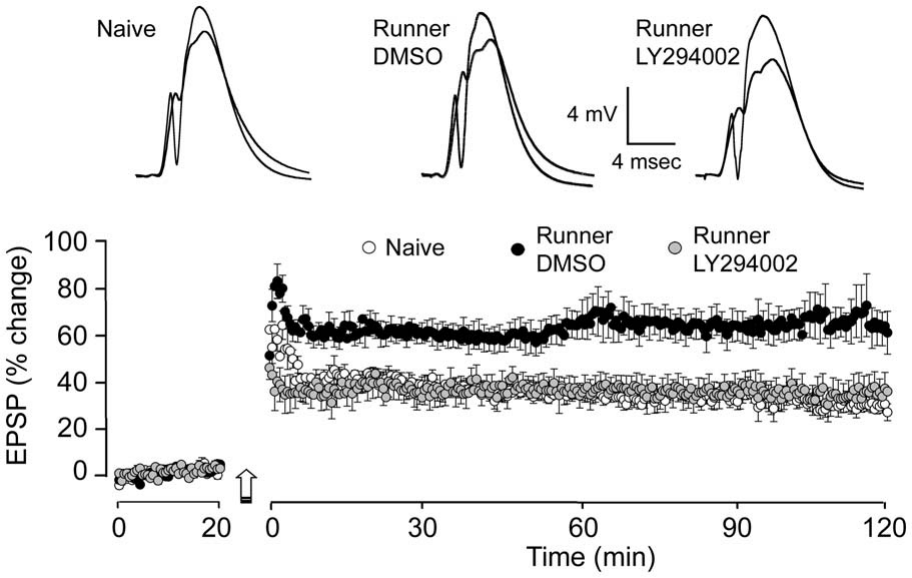
The effect of running on LTP in the dentate gyrus. The point plot represents the percent change in the EPSP recorded for 2 hours post tetanus (indicated by the arrow). Each point represent stimulated response every 30 sec with error bar on every second point. The mean of the last 15 minutes of recording showed 28.72±3.50% potentiation of the EPSP slope in naïve control rats (Grey diamonds, n = 8). In contrast, exercising rats infused with DMSO (Black circles, n = 7) show over 64.84±9.13% potentiation of the EPSP. Exercising rats infused with the PI3K inhibitor, LY294004 (Open circles, n = 7) show near identical potentiation of EPSP (33.26±6.69%) than that of naïve rats. Above are representative field potentials recorded before and after LTP induction in the three groups.

Previous studies have shown that the neurogenic effect of exercise is associated with an increased capacity for LTP in the dentate gyrus [13], [14], suggesting that the addition of young newborn neurons augments the capacity for plasticity in this structure. We wished to extend these findings and test the prediction that the reduction in the number of newborn neurons surviving two weeks after exercise caused by inhibiting PI3K-Akt signaling would suppress the beneficial effect of exercise on LTP. Thus, immediately before taking the brains for immunohistochemistry 14–16 days after the end of exercise, we examined LTP of the perforant path-to dentate granule cell synapses *in vivo* in the 3 groups. Induction of LTP in the dentate gyrus showed no overall difference between groups in short-term potentiation, measured across the first 5 min following the tetanus (F(1,9) = 0.285; p = 0.98). However there was a 2-fold increase in the magnitude of LTP of the EPSP in exercised rats infused with DMSO (57.47±6.58%; n = 7; Fig. 6) compared with naïve rats (28.72±2.87%; n = 8). And, importantly, although rats treated with LY294002 did show EPSP potentiation (37.71±6.51%; n = 8), this was comparable with that of the naïve rats and considerably less than in DMSO-treated rats (Fig. 6). Analysis of variance (F(2,20) = 5.27; p = 0.0003) and Fisher PLSD post hoc analyses confirmed the significant facilitation of LTP in DMSO-treated rats and the lack of facilitation in LY294002-treated rats (DMSO *vs* naïve and DMSO *vs* LY294002: p<0.05, LY294002 *vs* naïve: p>0.05). A similar pattern was observed with LTP of the population spike (Data not shown). Exercised DMSO-treated rats showed greater potentiation of the population spike (486.8±61.32%), compared with naïve (246.49±61.32%) and LY294002-treated rats (205.26±61.23%). The differences in the magnitude of LTP between groups was not due to differences in size of the baseline EPSP slope (F(2,20) = 0.038; p = 0.963), population spike amplitude (F(2,20) = 1.20; p = 0.132) or intensity of stimulation (F(2,20) = 0.535; p = 0.594). These data confirm that exercise results in facilitation of LTP in the dentate gyrus [13], [14] and show that despite no difference in the number of kilometers run, exercise-induced facilitation of LTP is abolished by inhibition of PI3K, reinforcing the premise that enhanced neurogenesis is directly related to the increased capacity for plasticity in the dentate gyrus [29].

## Discussion

Currently there is a great deal of effort being made to understand the potential cell-signaling mechanisms that drive experience-dependent neurogenesis in the adult brain. Certain regulators of proliferation and survival of newborn cells have been identified, that include growth factors and morphogens, hormones, certain neurotransmitters, intracellular signaling molecules and transcription factors [1]. Much of this knowledge however derives from studies in cell cultures, while the mechanisms associated with neurogenesis in the intact, behaving animal remain poorly defined. To date, it is clear that growth factors are necessary for neurogenesis in the behaving animal [30]–[31], and some studies have shown that genetic or pharmacological inactivation of growth-related molecules, such as VEGF, SDF-1, FGF-2, and IGF-1 implicated in neurogenesis can abrogate the beneficial effect of exercise or environmental enrichment on neurogenesis and on learning and memory or synaptic plasticity [1], [32]–[34].

The aim of the present experiments was to investigate the potential role of the PI3K-Akt signaling pathway in exercise-induced neurogenesis and its subsequent effect on LTP in the dentate gyrus as a functional readout. To this end, we used a protocol that was designed to examine both proliferation and later survival of neurons generated during the last three days of exercise. Our results firstly confirm that exercise (a) induces hyperphosphorylation of Akt [20], (b) promotes proliferation and survival of dentate gyrus cells [13]–[14]; and (c) facilitates the ability to induce LTP in the dentate gyrus [13]–[14]. Secondly, they show that icv infusion of the PI3K inhibitor, LY294002 abrogates exercise-induced phosphorylation of Akt and of several target proteins, survival of exercise generated newborn neurons and facilitation of LTP, but has no effect on cell proliferation following exercise.

The PI3K-Akt signaling pathway is known to be involved in a diverse range of cellular function [35]–[36] including all aspects of neurogenesis; cell cycle progression, migration and cell survival [36]–[37]. Being originally described as an oncogene, Akt was first known for its ability to promote cell survival by inhibiting cell death in numerous forms of cancer [37]. More recently, Akt's role in cell proliferation has been shown to be mediated by its interaction with proteins directly involved in cell cycle progression [38]. However Akt has also been shown to beneficially promote neurogenesis following brain injury via activation of the VEGF receptor [39].

Our results on exercise-induced hyperphosphorylation of Akt are in keeping with those of Chen and Russo-Neustadt [20]. In their study however, they showed that Akt was only partially activated at the threonine site only, and this was not sufficient for activating the downstream targets, FOXO1 and GSK3β. In our experiments we found exercise-induced phosphorylation of Akt at both Thr308 and Ser473 residues, a precondition for full activation of the protein [40); and consistent with this, we found a concomitant increase in phosphorylation of Akt's downstream targets, FOXO1, BAD and GSK3β after exercise. The difference between the two studies might well be due to greater specificity of the biochemical measures in sub-dissected dentate gyrus compared with the whole hippocampus, as Akt may be differentially phosphorylated by exercise in distinct hippocampal sub-regions.

Although it is not surprising that inhibition of PI3K blocked exercise-induced hyperphosphorylation of Akt and downstream protein targets it was surprising, given the suggestion that Akt modulates cell proliferation [38], [41] and is expressed in progenitors [42] that exercising rats infused with the PI3K inhibitor showed an identical increase in the numbers of proliferating cells as did the control runners. Exercise has been shown to regulate a number of growth factors such as IGF, FGF [21], [43], VEGF [44] and BDNF [45], that are associated with proliferation and cell survival; all of which can activate the PI3K-Akt signaling pathway. Two possible explanations for the lack of effect that blocking Akt has on proliferation is that (a) either exercise-driven proliferation in the dentate gyrus does not require activation of Akt, or (b) if it does play a potential role in proliferation, the effect of blocking phosphorylation if Akt may be compensated for by other proteins that are not directly associated with the PI3K-Akt signaling pathways. In either case, number of proteins, such as the cannabinoids [46], Sonic Hedgehog [47], WNT [48] and β-endorphin gyrus [49] are associated with proliferation in the dentate gyrus. Most notably, Koehl and colleagues [49] have shown that β-endorphin is necessary for exercise-induced proliferation in the dentate gyrus.

We also found that ERK, a kinase involved in cell growth and differentiation [50] that has been implicated in proliferation in the adult dentate gyrus [51]–[53] and is hyperphosphorylated in the hippocampus by exercise [18], [54]–[55], was not activated at the end of the exercise period in our experiments. Shen and colleagues [54] however have shown that exercise-induced regulation ERK occurs in a delay dependent manner, and it is possible in our experiments ERK may be regulated at a time point other than that we investigated.

In contrast to normal exercise-induced cell proliferation, the number of surviving BrdU-labeled cells 14 to 16 days later was significantly reduced in exercised rats infused with the PI3K inhibitor. The fact that there is an increase in cell proliferation at the end of the exercise period in both running groups, and that the majority of newborn neurons die by programmed cell death within the first week of generation [3], suggests that the PI3K-Akt signaling pathway is implicated in the promotion of survival of newborn cells following exercise by inactivating proapoptotic proteins. Akt is known to mediate its anti apoptotic function by negative regulation of Bcl-2 homology domain 3 only proteins, such as the FOXO transcription factors and BAD and inactivation of other proapoptotic proteins such as GSK3β. Akt phosphorylation of BAD and FOXO1 signals to 14-3-3 proteins bind to BAD to prevent it tethering the antiapoptotic proteins Bcl-2 and Bcl-X_L_ [56] and to FOXO1 which leads to its nuclear exclusion thereby preventing transcription of death associated genes [57]. GSK3β is principally associated with apoptosis via intrinsic mechanisms such as mitochondrial disruption and the priming the apoptotic process via regulating transcription factors and proteins associated with death pathways [58], and its phosphorylation by Akt functionally inactivates it. As these proteins induce apoptosis in the mitochondria and the nucleus, it would suggest that Akt mediates survival of newborn cells in a cell autonomous manner [36], [59], around the time of neuron birth and/or during the two weeks following exercise. Evidence has shown (a) a delay of 2.5 day of a neuroprotective response by Akt to apoptotic injury in cell cultures [60] and (b) a prolonged elevation of BDNF levels by 7 days following exercise [61]. This suggests that survival signals can respond in both a delayed and prolonged manner. However, the precise temporal window during which Akt-mediated pro-survival signals are required to prevent neurons from dying remains to be investigated.

Although blocking PI3K-mediated activation of Akt and its downstream targets by infusion of LY294002 drastically reduced survival of newborn neurons generated by exercise, it did not completely block neurogenesis, suggesting that other proteins are implicated in the survival of newborn neurons. It is known to date that a number of proteins, including neurotransmitters, hormones, signaling molecules can influence the neurogenic process [62]–[63] and some of these have also been shown to be regulated by exercise, although the link between exercise and neurogenesis has not been made. In addition, although Akt phosphorylates BAD, FOXO1 and GSK3β, it is not the sole activator of these proteins; members of the MAPK/ERK signaling pathway and other kinases, such as p70S6 kinase, p90Rsk, certain isoforms of PKC and PKA are able to phosphorylate BAD and GSK3β [18], [64]–[66]. Therefore, there are a number of prime candidates that may well contribute to exercise-induced neurogenesis. Nonetheless, our results suggest that exercise-induced neurogenesis and the consequential facilitation of plasticity in the dentate gyrus strongly relies on functional activation of PI3K-Akt prosurvival pathways via inactivation of proapoptotic target proteins. Consistent with this, an increase in survival of adult dentate gyrus progenitor cells has been reported in mice overexpressing the anti-apoptotic protein Bcl-2 [67] as well as mice deficient for the pro-apoptotic protein Bax [68].

Finally, the decrease in survival of exercise-generated cells in the dentate gyrus was accompanied by suppression of exercise-induced facilitation of LTP. Although within the time window of 14–16 days of age, surviving neurons are considered relatively immature, they already harbor functional synaptic connections and have unique physiological properties that suggest they may contribute to the increased capacity for plasticity in the dentate gyrus and the facilitation of certain forms of memory [1], [15], [69]. This would suggest that activation of the PI3K-Akt signaling pathway is an important mechanism contributing to the survival of newborn cells stimulated by exercise, that are capable of bestowing facilitation on synaptic plasticity in the dentate gyrus.

In conclusion, the data we present here suggest that the survival of newborn cells generated in the dentate gyrus by exercise requires the functional activation of the PI3K-Akt signalling pathway, whereas it is not essential for cell proliferation. Although a number of studies have shown that Akt does play a role in proliferation, many of these studies have been expressly designed to investigate its role in carcinogenic processes within a malignant cellular environment. Our results are more in keeping with the suggestion that certain trophic factors may be more instrumental in regulating proliferation, whereas other mechanisms that trigger the activation of intracellular signaling cascades would promote survival [70]. As to how Akt promotes the survival of these cells we suggest is via its ability to inactivate proapoptotic target proteins. Although we suggest that activation of this signalling pathway provides a signal for the promotion of survival of newborn cells in the dentate gyrus generated during exercise, it is by no means the only signalling cascade implicated in the process of neurogenesis; as suggested by the remaining level of surviving cells in LY294002-treated rats. Currently our understanding of the cellular and molecular mechanisms underlying the different stages of environmentally regulated neurogenesis is meagre. The present findings highlight the contribution of the PI3K-Akt pathway in maintaining experience-dependent neurogenesis in the adult dentate gyrus, presenting a first step towards gaining a more in depth understanding of mechanisms associated with the beneficial effects of neurogenesis *in vivo*.

## References

[1] Zhao C, Deng W, Gage FH (2008). Mechanisms and functional implications of adult neurogenesis.. Cell.

[2] van Praag H, Kempermann G, Gage FH (1999a). Running increases cell proliferation and neurogenesis in the adult mouse dentate gyrus.. Nat Neurosci.

[3] Kempermann G, Gast D, Kronenberg G, Yamaguchi M, Gage FH (2003). Early determination and long-term persistence of adult-generated new neurons in the hippocampus of mice.. Dev Dis.

[4] Van Praag H, Kempermann G, Gage FH (2000). Neural consequences of environmental enrichment.. Nat Rev Neurosci.

[5] Bruel-Jungerman E, Laroche S, Rampon C (2005). New neurons in the dentate gyrus are involved in the expression of enhanced long-term memory following environmental enrichment.. Eur J Neurosci.

[6] Hillman CH, Motl RW, Pontifex MB, Posthuma D, Stubbe JH (2006). Physical activity and cognitive function in a cross-section of younger and older community-dwelling individuals.. Heal Psychol.

[7] Kramer AF, Erickson KI (2007). Capitalizing on cortical plasticity: influence of physical activity on cognitive and brain function.. Trends Cog Sci.

[8] Kramer AF, Erickson KI, Colcombe SJ (2006). Exercise, cognition, and the aging brain.. J Appl Physiol.

[9] Hillman CH, Erickson KI, Kramer AF (2008). Be smart, exercise your heart: exercise effects on brain and cognition.. Nat Rev Neurosci.

[10] Morgan D (2007). Amyloid, memory and neurogenesis.. Expl Neurol.

[11] Vaynman S, Ying Z, Gomez-Pinilla F (2004). Hippocampal BDNF mediates the efficacy of exercise on synaptic plasticity and cognition.. Eur J Neurosci.

[12] Schweitzer NB, Alessio HM, Berry SD, Roeske K, Hagerman AE (2006). Exercise-induced changes in cardiac gene expression and its relation to spatial maze performance.. Neurochem Int.

[13] van Praag H, Christie BR, Sejnowski T, Gage FH (1999b). Running enhances neurogenesis, learning and long-term potentiation in mice.. Proc Natl Acad Sci USA.

[14] Farmer J, Zhao X, van Praag H, Wodke K, Gage FH (2004). Effects of voluntary exercise on synaptic plasticity and gene expression in the dentate gyrus of adult male Sprague Dawley rats in vivo.. Neuroscience.

[15] Schmidt-Hieber C, Jonas P, Bischofberger J (2004). Enhanced synaptic plasticity in newly generated granule cells of the adult hippocampus.. Nature.

[16] Ming GL, Song H (2005). Adult neurogenesis in the mammalian central nervous system.. Annu Rev Neurosci.

[17] Tong L, Shen H, Perreau VM, Balazs R, Cotman CW (2003). Effects of exercise on gene-expression profile in the rat hippocampus.. Neurobiol Dis.

[18] Ding Q, Vaynman S, Akhavan M, Ying Z, Gomez-Pinilla F (2006). Insulin-like growth factor 1 interfaces with brain-derived neurotrophic factor mediated synaptic plasticity to modulate aspects of exercise-induced cognitive function.. Neuroscience.

[19] Cotman CW, Berchtold NC, Christie LA (2007). Exercises builds brain health: key roles of growth factor cascades and inflammation.. Trends Neurosci.

[20] Chen M, Russo-Neustadt AA (2005). Exercise activates the phosphatidylinositol 3-kinase pathway.. Mol Brain Res.

[21] Aberg MA, Aberg ND, Palmer TD, Aborn AM, Carlsson-Skwirut C (2003). IGF-1 has a direct proliferative effect in adult hippocampal progenitor cells.. Mol Cell Neurosci.

[22] Brazil DP, Yang ZZ, Hemmings BA (2004). Advances in protein kinase B signalling: AKTion on multiple fronts.. Trends Biochem Sci,.

[23] Davis S, Vanhoutte P, Pages C, Caboche J, Laroche S (2000). The MAPK/ERK cascade targets both Elk-1 and cAMP response element-binding protein to control long-term potentiation-dependent gene expression in the dentate gyrus in vivo.. J Neurosci.

[24] Horwood JM, Dufour F, Laroche S, Davis S (2006). Signalling mechanisms mediated by the phosphoinositide 3-kinase/Akt cascade in synaptic plasticity and memory in the rat.. Eur J Neurosci.

[25] Bruel-Jungerman E, Davis S, Rampon C, Laroche S, Rampon C (2006). Long-term potentiation enhances neurogenesis in the adult dentate gyrus.. J Neurosci.

[26] Kelly A, Laroche S, Davis S (2003). Activation of mitogen-activated protein kinase/extracellular signal-regulated kinase in hippocampal circuitry is required for consolidation and reconsolidation of recognition memory.. J Neurosci.

[27] Kee N, Sivalingam S, Boonstra R, Wojtowicz JM (2002). The utility of Ki-67 and BrdU as proliferative markers of adult neurogenesis.. J Neurosci Meths.

[28] Endl E, Gerdes J (2000). The ki-67 protein: fascinating forms and an unknown function.. Exp Cell Res.

[29] Snyder JS, Kee N, Wojtowicz JM (2001). Effects of adult neurogenesis on synaptic plasticity in the rat dentate gyrus.. J Neurophysiol.

[30] Hagg T (2005). Molecular regulation of adult CNS neurogenesis: and integrated view.. Trends Neurosci.

[31] Galvan V, Greenberg DA, Jin K (2006). The role of vascular endothelial growth factor in neurogenesis in adult brain.. Mini Rev Med Chem.

[32] Cao L, Jiao X, Zuzga DS, Liu Y, Fong DM (2004). VEGF links hippocampal activity with neurogenesis, learning and memory.. Nat Genet.

[33] Kolodziej A, Schulz S, Guyon A, Wu DF, Pfeiffer M (2008). Tonic activation of CXC chemokine receptor 4 in immature granule cells supports neurogenesis in the adult dentate gyrus.. J Neurosci.

[34] Trejo JL, Llorens-Martin MV, Torres-Aleman I (2008). The effect of exercise on spatial learning and anxiety-like behaviour are mediated by an IGF-1-dependent mechanism related to hippocampal neurogenesis.. Mol Cell Neurosci.

[35] Brazil DP, Hemmings BA (2001). Ten years of protein kinase B signaling: a hard Akt to follow.. Trends Biochem Sci.

[36] Wymann MP, Zvelebil M, Laffargue M (2003). Phosphoinositide 3-kinase signaling – which way to target?. Trends Neurosci.

[37] Hill MM, Hemmings BA (2002). Inhibition of protein kinase B/Akt: implications for cancer therapy.. Pharmacol Ther.

[38] Manning BD, Cantley LC (2007). AKT/PKB signalling: navigation downstream.. Cell.

[39] Wu H, Lu D, Jiang H, Xiong Y, Qu C (2008). Simvastatin-mediated upregulation of VEGF and BDNF, activation of th PI3K/Akt pathway, and increase of neurogenesis are associated with therapeutic improvement after traumatic brain injury.. J Neurotrauma.

[40] Alessi DR, Andjelkovik M, Caudwell FB, Cron P, Morrice N (1996). Mechanisms of activation of protein kinase B by insulin and IGF-1.. EMBO J.

[41] Woodgett JR (2005). Recent advances in the protein kinase B signaling pathway.. Curr Opin Cell Biol.

[42] Sinor AD, Lillien L (2004). Akt-1 expression levels regulates CNS precursors.. J Neurosci.

[43] Trejo JL, Carro E, Torres-Aleman I (2001). Circulating insulin-like growth factor 1 mediates exercise-induced increases in the number of new neurons in the adult hippocampus.. J Neurosci.

[44] Fabel K, Fabel K, Tam B, Kaufer D, Baiker A (2003). VEGF is necessary for exercise-induced adult hippocampal neurogenesis.. Eur J Neurosci.

[45] Lee E, Son H (2009). Adult hippocampal neurogenesis and related neurotrophic factors.. BMB Rep.

[46] Galve-Roperh I, Aquado T, Palazuelos J, Guzman M (2007). The endocannabinoid system and neurogenesis in health and disease.. Neuroscientist,.

[47] Lai K, Kaspar BK, Gage FH, Schaffer DV (2003). Sonic hedgehog regulates neural progenitor cell proliferation in vitro and in vivo.. Nat Neurosci.

[48] Lie DC, Colamarino SA, Song HJ, Désiré L, Mira H (2005). Wnt signaling regulates adult hippocampal neurogenesis.. Nature.

[49] Koehl M, Meerlo P, Gonzales D, Rontal A, Turek FW (2008). Exercise-induced promotion of hippocampal cell proliferation requires beta-endorphin.. FASEB J.

[50] Shaul YD, Seger R (2007). The MEK/ERK cascade: from signaling specificity to diverse functions.. Biochim Biophys Acta.

[51] Yan XB, Hou HL, Wu LM, Liu J, Zhou JN (2007). Lithium regulates hippocampal neurogenesis by ERK pathway and facilitates recovery of spatial learning and memory in rats after transient global cerebral ischemia.. Neuropharmacol.

[52] Choi YS, Cho HY, Hoyt KR, Naegele JR, Obrietan K (2008). GF-1 receptor-mediated ERK/MAPK signaling couples status epilepticus to progenitor cell proliferation in the subgranular layer of the dentate gyrus.. Glia,.

[53] Kim SJ, Son TG, Park HR, Park M, Kim MS (2008). Curcumin stimulates proliferation of embryonic neural progenitor cells and neurogenesis in the adult hippocampus.. J Biol Chem.

[54] Shen H, Tong L, Balazs R, Cotman CW (2001). Physical activity elicits sustained activation of the cyclic AMP response element-binding protein and mitogen-activated protein kinase in the rat hippocampus.. Neuroscience.

[55] Muller AP, Cammarota M, Dietrich MO, Rotta LN, Portela LV (2008). Different effect of high fat diet and physical exercise in the hippocampal signaling.. Neurochem Res.

[56] Datta SR, Dudek H, Tao X, Masters S, Fu H (1997). Akt phosphorylation of BAD couples survival signals to the cell-intrinsic death machinery.. Cell.

[57] Brunet A, Bonni A, Zigmond MJ, Lin MZ, Juo P (1999). Akt promotes cell survival by phosphorylating and inhibiting a Forkhead transcription factor.. Cell.

[58] Beurel E, Jope RS (2008). The paradoxical pro-and anti-apoptotic actions of GSK3 in the intrinsic and extrinsic apoptosis signalling pathways.. Prog Neurobiol.

[59] Parcellier A, Tintignac LA, Zhuravleva E, Hemmings BA (2008). PKB and the mitochondria: AKTing on apoptosis.. Cell Signaling.

[60] Park SM, Jung JS, Jang MS, Kang KS, Kang SK (2008). Transforming growth factor-β1 regulates the fate of cultured spinal cord-derived neural progenitor cells.. Cell Prolif.

[61] Berchtold NC, Chinn G, Chou M, Kesslak JP, Cotman CW (2005). Exercise primes a molecular memory for brain derived neurotrophic factor protein induced in the rat hippocampus.. Neuroscience.

[62] Abrous DN, Koehl M, Le Moal M (2005). Adult neurogenesis: from precursor to network and physiology.. Physiol Rev.

[63] Lledo PM, Alonso M, Grubb MS (2006). Adult neurogenesis and functional plasticity in neuronal circuits.. Nat Rev Neurosci,.

[64] Grimes CA, Jope RS (2001). The multifaceted roles of glycogen synthase kinase 3β in cellular signalling.. Prog Neurobiol.

[65] Jin K, Mao XO, Zhu Y, Greenberg DA (2002). MEK and ERK protect hypoxic cortical neurons via phosphorylation of BAD.. J Neurochem.

[66] Ho KK, Myatt SS, Lam EW-F (2008). Many forks in the path: cycling with FoxO.. Oncogene.

[67] Kuhn HG, Biebl M, Wilhelm D, Li M, Friedlander RM (2005). Increased generation of granule cells in adult Bcl-2 overexpressing mice: a role for cell death during continued hippocampal neurogenesis.. Eur J Neursci.

[68] Sun W, Winseck A, Vinsant S, Park OH, Kim H (2004). Programmed cell death of adult-generated hippocampal neurons is mediated by the proapoptotic gene Bax.. J Neurosci.

[69] Ge S, Yang CH, Hsu KS, Ming GL, Song H (2007). A critical period for enhanced synaptic plasticity in newly generated neurons of the adult brain.. Neuron.

[70] Olson AK, Eadie BD, Ernst C, Christie BR (2006). Environmental enrichment and voluntary exercise massively increase neurogenesis in the adult hippocampus via dissociable pathways.. Hippocampus.

